# Tuberculous Cervical Lymphadenitis (Scrofula) Associated with Massive Pleural Effusion in an Immunocompetent Adult: A Rare Presentation of Extrapulmonary Tuberculosis Confirmed by GeneXpert MTB/RIF

**DOI:** 10.3390/diagnostics16193249

**Published:** 2026-10-08

**Authors:** Sergiu Ioan Murg, Maria Daniela Moț, Flavius Ioan Moț, Andreea Sorina Joldea, Laura Ioana Rădoi, Florin Mihai Șandor, Ana-Liana Bouros-Tătaru, Cristina Georgiana Firu, Denis Bogdan Butari, Paula Irina Barata

**Affiliations:** 1 Department of Oncology, Faculty of Medicine, “Vasile Goldis” Western University of Arad, Blvd. Revolutiei, No. 96, 310025 Arad, Romania; murg.sergiu@uvvg.ro; 2 Department of General Medicine, “Vasile Goldis” Western University of Arad, Blvd. Revolutiei, No. 96, 310025 Arad, Romania; mot.daniela@uvvg.ro; 3 “Victor Babes” University of Medicine and Pharmacy Timisoara, Eftimie Murgu Square 2, 300041 Timisoara, Romania; motflaviusioan@gmail.com; 4 Department of Physiology, Faculty of Medicine, “Vasile Goldis” Western University of Arad, Blvd. Revolutiei, No. 96, 310025 Arad, Romania; joldea.andreea@uvvg.ro; 5 Department of Biology and Life Sciences, Faculty of Medicine, “Vasile Goldiș” Western University of Arad, Blvd. Revolutiei, No. 96, 310025 Arad, Romania; bondar.laura@uvvg.ro (L.I.R.); butari.denis-bogdan@uvvg.ro (D.B.B.); 6 Department of Heamatology, Faculty of Medicine, “Vasile Goldis” Western University of Arad, Bulevardul Revolutiei 94, 310025 Arad, Romania; firu.cristina@uvvg.ro; 7 Department of Pneumology, Faculty of Medicine, “Vasile Goldis” Western University of Arad, Blvd. Revolutiei, No. 96, 310025 Arad, Romania; barata.paula@uvvg.ro

**Keywords:** scrofula, tuberculous cervical lymphadenitis, extrapulmonary tuberculosis, pleural tuberculosis, GeneXpert MTB/RIF, pleural effusion, necrotizing granulomatous inflammation

## Abstract

**Background**: Tuberculous cervical lymphadenitis (scrofula) is the most common form of extrapulmonary tuberculosis, although its coexistence with massive pleural effusion is uncommon and may delay diagnosis because of its nonspecific clinical presentation. We report the case of a 47-year-old man presenting with a large right-sided pleural effusion associated with a suppurative cervical lymphadenitis, in whom rapid molecular testing established the diagnosis of disseminated extrapulmonary tuberculosis. **Case Presentation**: A 47-year-old male presented with progressive right-sided chest pain, exertional dyspnea, intermittent fever, night sweats, weight loss, and a progressively enlarging left laterocervical mass. Physical examination revealed multiple enlarged, fluctuant cervical lymph nodes and clinical signs of a massive right pleural effusion. Thoracic computed tomography demonstrated a large right pleural effusion with compressive atelectasis and enlarged cervical and mediastinal lymph nodes. Ultrasound-guided cervical lymph node drainage and tube thoracostomy were performed, followed by repeated pleural fluid evacuations through the chest tube. Purulent material obtained from the cervical abscess and pleural fluid were subjected to microbiological, cytological, and molecular investigations, including Xpert MTB/RIF (GeneXpert MTB/RIF). GeneXpert MTB/RIF detected *Mycobacterium tuberculosis* complex in both the drained cervical purulent material and pleural fluid, with no evidence of rifampicin resistance. Conventional bacterial cultures remained negative, while mycobacterial cultures were subsequently positive for *M. tuberculosis*. Pleural fluid analysis was consistent with an exudative lymphocytic effusion with elevated adenosine deaminase levels. Histopathological examination of the cervical tissue demonstrated necrotizing granulomatous inflammation with central caseous necrosis, epithelioid histiocytes, and Langhans-type multinucleated giant cells, supporting the diagnosis of tuberculous lymphadenitis. HIV serology was negative, and no additional immunodeficiency was identified. Standard first-line antituberculous therapy (isoniazid, rifampicin, pyrazinamide, and ethambutol) was initiated promptly following microbiological confirmation, leading to progressive regression of the cervical lesions, complete resolution of the pleural effusion, and favorable clinical evolution during follow-up. **Conclusions**: The simultaneous occurrence of tuberculous cervical lymphadenitis and massive pleural tuberculosis represents an uncommon presentation of extrapulmonary tuberculosis that requires a high index of clinical suspicion. The combination of histopathological examination and rapid molecular testing of extrapulmonary specimens allowed definitive and timely confirmation of tuberculosis and facilitated prompt initiation of appropriate therapy. Clinicians should consider tuberculosis in patients presenting with cervical suppurative lymphadenitis and unexplained pleural effusion, even in the absence of microbiologically confirmed pulmonary disease, to avoid diagnostic delay and improve clinical outcomes.

## 1. Introduction

Tuberculosis (TB), caused by *Mycobacterium tuberculosis*, remains one of the leading infectious causes of morbidity and mortality worldwide despite the availability of effective antimicrobial therapy for more than half a century. According to the World Health Organization (WHO) Global Tuberculosis Report 2025, an estimated 10.8 million people developed tuberculosis in 2024, and more than one million deaths were attributed to the disease, highlighting its continued global public health impact. Although pulmonary tuberculosis accounts for the majority of cases, extrapulmonary tuberculosis (EPTB) represents approximately 15–25% of all notified TB cases in immunocompetent individuals and up to 50% among patients with impaired cellular immunity [1,2].

Despite a continuous decline in tuberculosis incidence across Europe, EPTB remains an important clinical and diagnostic challenge. According to the European Centre for Disease Prevention and Control (ECDC) and the WHO Regional Office for Europe, extrapulmonary disease accounts for approximately one-fifth of all reported TB cases in the European Union and European Economic Area, with lymph node involvement being the most common presentation. The epidemiology of EPTB has evolved over recent decades because of increasing migration from high-burden countries, population aging, widespread use of immunosuppressive therapies, and improved diagnostic capabilities. Nevertheless, delayed diagnosis remains frequent because extrapulmonary manifestations are often nonspecific and mimic malignant, inflammatory, or other infectious diseases. These factors continue to contribute to significant morbidity despite well-established tuberculosis control programs throughout Europe [3,4,5].

Tuberculous cervical lymphadenitis, historically referred to as scrofula, is the most frequent form of extrapulmonary tuberculosis, accounting for approximately 30–40% of all EPTB cases. The disease results from lymphatic or hematogenous dissemination of *Mycobacterium tuberculosis* and is characterized by chronic granulomatous inflammation of the cervical lymph nodes. Patients typically present with slowly enlarging, painless cervical masses that progressively undergo caseous necrosis, liquefaction, abscess formation, and occasionally spontaneous cutaneous fistulization. Because of its indolent progression and variable clinical presentation, scrofula is frequently mistaken for bacterial lymphadenitis, lymphoma, metastatic head and neck malignancies, sarcoidosis, or other granulomatous disorders, frequently delaying definitive diagnosis and treatment [6,7,8].

Pleural tuberculosis is another common manifestation of extrapulmonary TB and represents one of the leading causes of exudative pleural effusion worldwide. It usually develops following rupture of a subpleural caseous focus into the pleural cavity or as a delayed hypersensitivity reaction to mycobacterial antigens. Patients commonly present with unilateral pleuritic chest pain, dyspnea, fever, and pleural effusion of variable volume. Massive pleural effusion, however, is relatively uncommon, while the coexistence of tuberculous cervical lymphadenitis and extensive pleural disease is reported only sporadically in the literature. Such presentations may be particularly challenging because respiratory symptoms may predominate, masking the underlying extrapulmonary dissemination [9,10].

The diagnosis of extrapulmonary tuberculosis remains difficult because most extrapulmonary specimens contain a low bacillary load, reducing the sensitivity of conventional acid-fast bacilli smear microscopy and even mycobacterial culture. Histopathological examination demonstrating necrotizing granulomatous inflammation with caseous necrosis remains an important diagnostic tool; however, microbiological confirmation is essential whenever possible. During the last decade, nucleic acid amplification techniques, particularly the Xpert MTB/RIF (GeneXpert MTB/RIF) assay, have significantly improved the diagnostic approach to extrapulmonary tuberculosis by enabling rapid identification of *M. tuberculosis* complex deoxyribonucleic acid (DNA) together with simultaneous detection of rifampicin resistance within a few hours. The use of GeneXpert on lymph node aspirates has shown excellent diagnostic performance, while its application to pleural fluid, although less sensitive, may provide rapid microbiological confirmation when interpreted in conjunction with clinical, radiological, and histopathological findings [11,12,13].

Herein, we present the case of a 47-year-old immunocompetent man with tuberculous cervical lymphadenitis (scrofula) associated with massive right-sided tuberculous pleural effusion, in whom *Mycobacterium tuberculosis* was rapidly identified by GeneXpert MTB/RIF in both cervical abscess material obtained after surgical drainage and pleural fluid obtained by thoracic drainage. To our knowledge, reports describing simultaneous molecular confirmation of tuberculosis from two distinct extrapulmonary sites in patients presenting with scrofula and massive pleural effusion remain uncommon. This case highlights the importance of maintaining a high index of suspicion for tuberculosis in patients presenting with suppurative cervical lymphadenopathy and unexplained pleural effusion and emphasizes the pivotal role of rapid molecular diagnostics in establishing an early diagnosis, guiding timely initiation of antituberculous therapy, and improving patient outcomes.

## 2. Case Report

This case report describes the diagnostic work-up, therapeutic management, and clinical outcome of a patient with concomitant tuberculous cervical lymphadenitis (scrofula) and massive right-sided pleural tuberculosis. The report was prepared in accordance with the CARE (CAse REport) guidelines [11] to ensure transparent and comprehensive reporting of clinical findings.

A 47-year-old man was admitted to the Department of Pulmonology, Arad County Emergency Clinical Hospital, Romania, with a three-week history of progressive dyspnea, right-sided pleuritic chest pain, persistent dry cough, intermittent fever, night sweats, fatigue, and an enlarging painful swelling in the left cervical region. The patient denied previous tuberculosis treatment, recent travel, or known contact with active tuberculosis. His medical history was otherwise unremarkable, and no immunosuppressive conditions or chronic diseases were identified.

Physical examination revealed decreased breath sounds and dullness to percussion over the entire right hemithorax, consistent with a massive pleural effusion. A fluctuant laterocervical mass measuring approximately 5 × 4 cm with overlying inflammatory skin changes was also observed, suggestive of suppurative cervical lymphadenitis.

The initial laboratory evaluation included complete blood count, inflammatory markers, liver and renal function tests, coagulation profile, HIV serology, and routine biochemical investigations.

Chest radiography and contrast-enhanced thoracic computed tomography (CT) were performed to assess the extent of thoracic involvement. Cervical ultrasonography was used to characterize the lymph node lesion and to guide surgical drainage.

A chest tube was inserted because of the large right pleural effusion, allowing pleural fluid sampling and therapeutic drainage. Subsequent pleural fluid evacuations were performed through the chest tube.

Simultaneously, surgical incision and drainage of the cervical abscess were performed. Purulent material and tissue fragments obtained during the procedure were submitted for histopathological examination, Gram staining, Ziehl–Neelsen staining, bacterial culture, mycobacterial culture, and Xpert MTB/RIF (GeneXpert MTB/RIF) molecular analysis.

The Xpert MTB/RIF assay (Cepheid, Sunnyvale, CA, USA) was performed according to the manufacturer’s recommendations on both pleural fluid and cervical abscess specimens to detect *Mycobacterium tuberculosis* complex DNA and rifampicin resistance.

Samples were simultaneously inoculated onto liquid Mycobacterial Growth Indicator Tube (MGIT) 960 and solid Löwenstein–Jensen media for mycobacterial culture. Conventional aerobic bacterial cultures were also performed to exclude pyogenic infection.

Histopathological examination of cervical tissue specimens included hematoxylin and eosin staining, with microscopic evaluation for granulomatous inflammation, caseous necrosis, and multinucleated Langhans giant cells.

Following microbiological confirmation of tuberculosis, standard first-line antituberculous therapy consisting of isoniazid, rifampicin, pyrazinamide, and ethambutol was initiated according to current WHO recommendations. Therapeutic response was monitored through serial clinical evaluations, laboratory investigations, and follow-up chest imaging. The patient’s clinical course, resolution of pleural effusion, regression of cervical lymphadenopathy, and treatment tolerance were documented throughout hospitalization and subsequent outpatient follow-up.

### 2.1. Clinical Presentation

A 47-year-old man was admitted to the Department of Pulmonology with progressive dyspnea, right-sided pleuritic chest pain, nonproductive cough, intermittent fever, night sweats, fatigue, and a painful swelling in the left cervical region that had progressively enlarged over the preceding three weeks. The patient denied previous tuberculosis, recent hospitalization, or known exposure to individuals with active tuberculosis.

**Figure 1:**
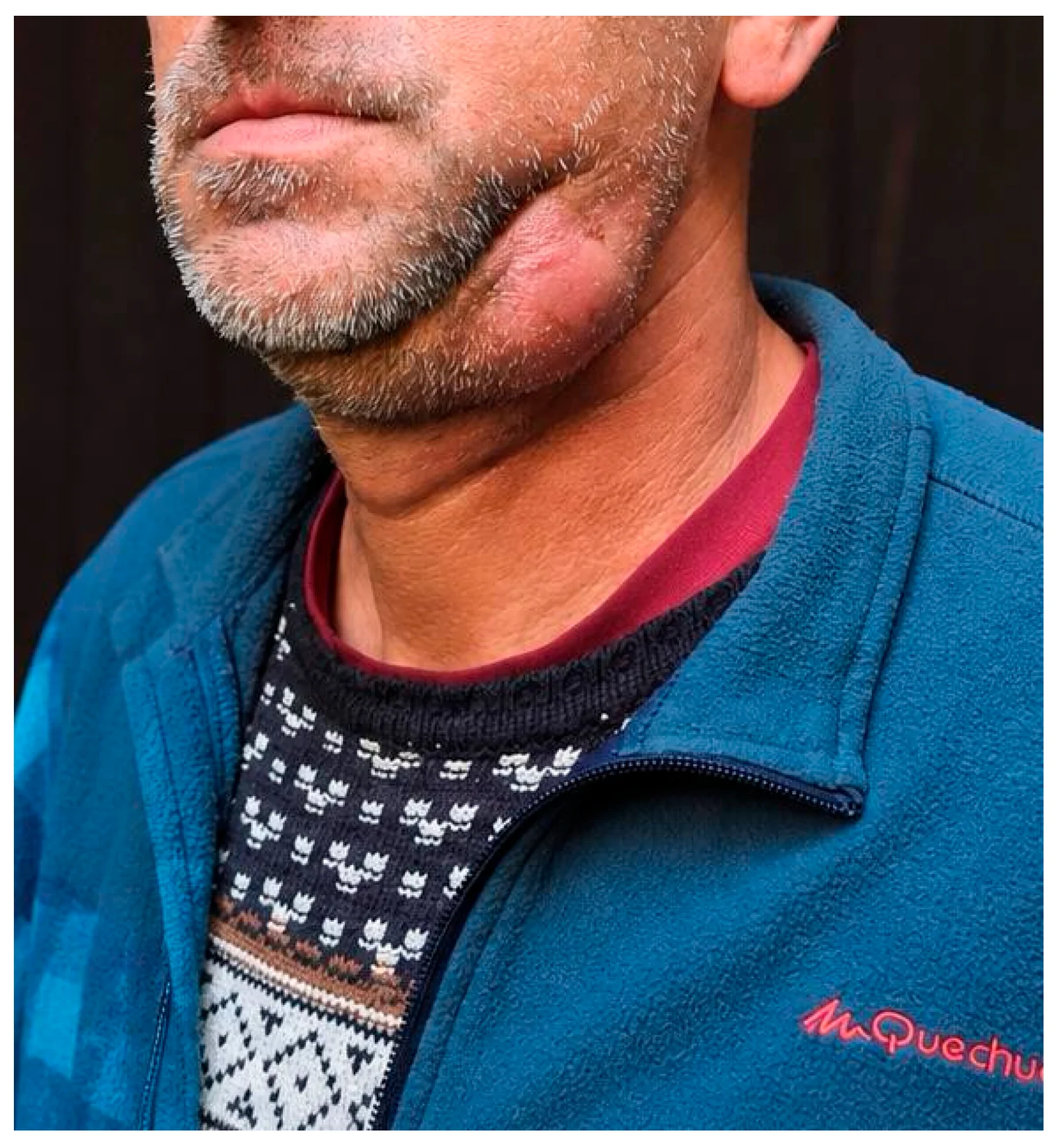
Cervical abscess formation.

On admission, the patient was hemodynamically stable but mildly tachypneic. Physical examination revealed markedly decreased breath sounds and dullness to percussion over the right hemithorax, suggestive of a large pleural effusion. In addition, a fluctuant, tender laterocervical mass measuring approximately 5 × 4 cm was identified, with local inflammatory changes consistent with cervical abscess formation (Figure 1).

### 2.2. Laboratory and Imaging Findings

Initial laboratory investigations revealed a significant inflammatory response, with a C-reactive protein level of 96.4 mg/L and an erythrocyte sedimentation rate of 84 mm/h. The white blood cell count was mildly elevated at 10.4 × 10^9^/L, with neutrophils accounting for 72% of leukocytes. Thrombocytosis was present, with a platelet count of 468 × 10^9^/L, while mild anemia was observed, with a hemoglobin level of 11.8 g/dL. Serum albumin was decreased to 3.2 g/dL, and lactate dehydrogenase was elevated at 382 U/L. Renal and hepatic function were preserved, with a creatinine level of 0.89 mg/dL, aspartate aminotransferase (AST) of 28 U/L, and alanine aminotransferase (ALT) of 31 U/L. Procalcitonin was within the reference range (0.18 ng/mL), and HIV serology was negative. The main laboratory findings are summarized in Table 1.

**Figure 2:**
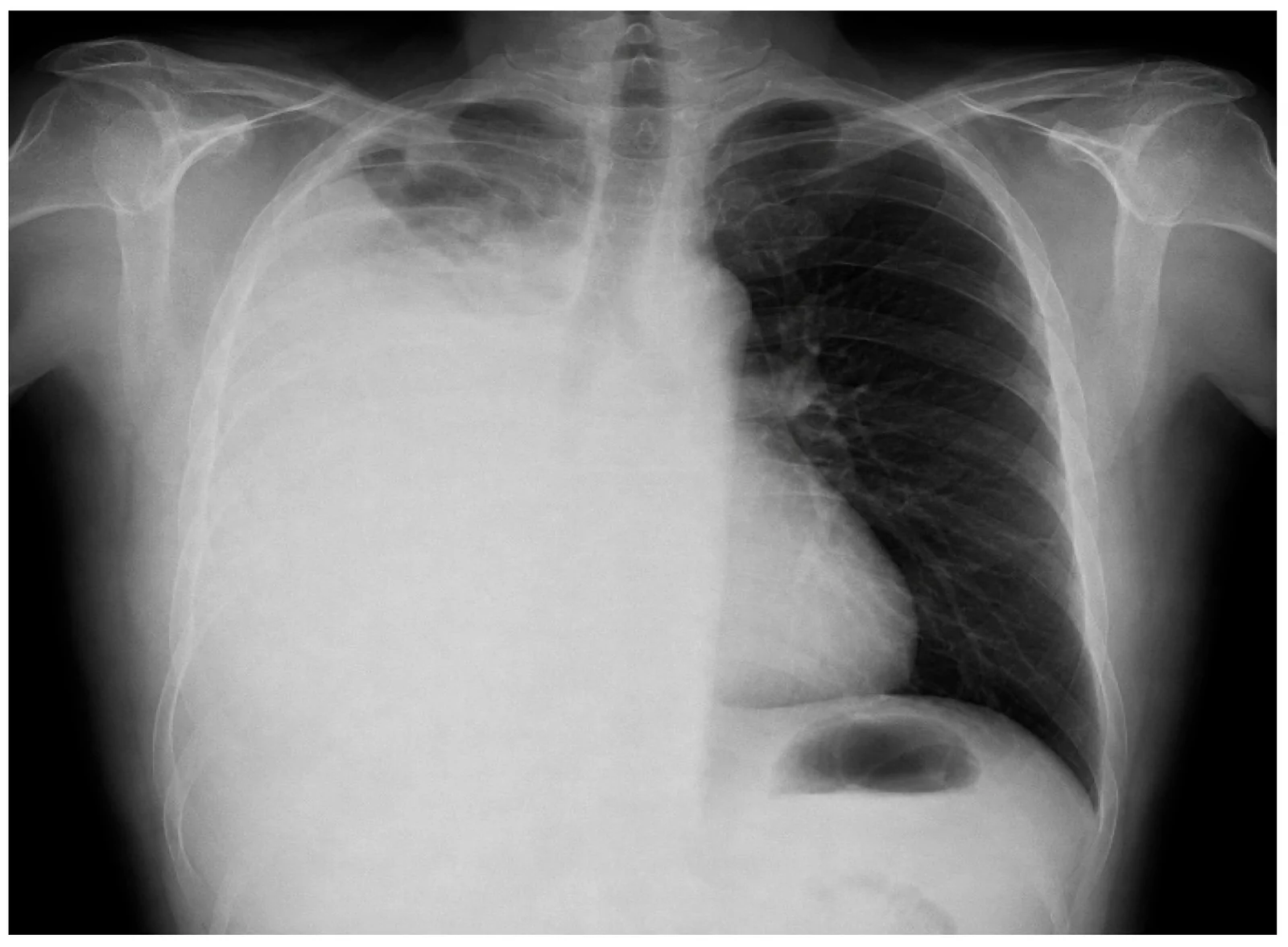
Chest radiography showing complete opacification of the lower two-thirds of the right hemithorax due to a massive right-sided pleural effusion.

Chest radiography demonstrated complete opacification of the lower two-thirds of the right hemithorax. Contrast-enhanced thoracic computed tomography confirmed a massive right-sided pleural effusion associated with compressive atelectasis of the adjacent lung parenchyma. Enlarged cervical and mediastinal lymph nodes were also identified, without radiological evidence of cavitary pulmonary lesions (Figure 2).

**Figure 3:**
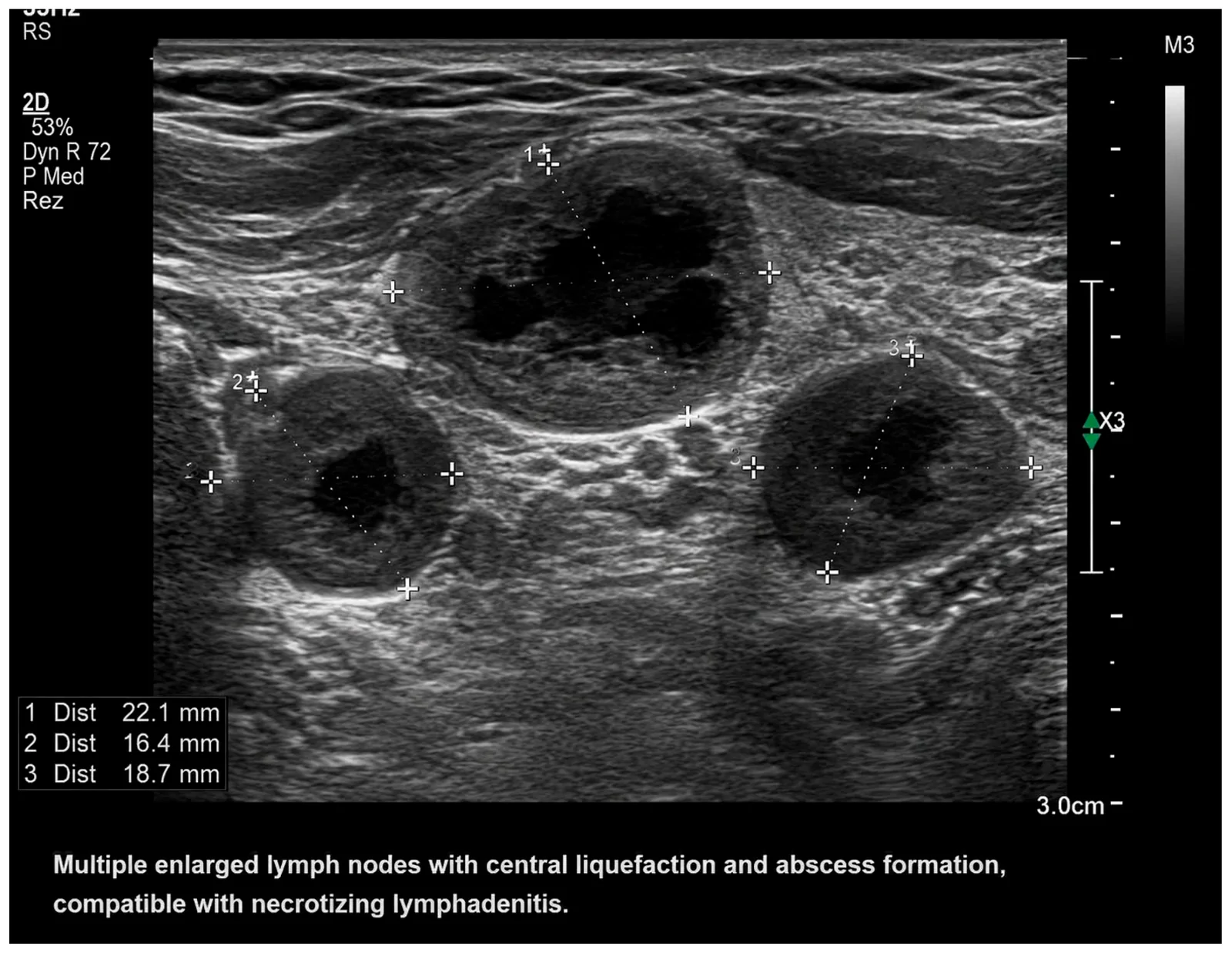
Cervical ultrasonography.

Cervical ultrasonography demonstrated multiple enlarged lymph nodes with central liquefaction and abscess formation, compatible with necrotizing lymphadenitis (Figure 3).

### 2.3. Microbiological and Histopathological Findings

Because of the significant pleural effusion, tube thoracostomy was performed, allowing evacuation of a large volume of straw-colored pleural fluid. The chest tube was subsequently used for repeated pleural fluid drainage procedures during the following 41 days. Simultaneously, the cervical abscess was surgically drained, yielding abundant purulent material.

Pleural fluid analysis revealed an exudative, lymphocyte-predominant effusion, with a protein concentration of 5.2 g/dL, LDH of 684 U/L, glucose of 78 mg/dL, and pH of 7.34. The adenosine deaminase (ADA) level was elevated at 87 U/L. The total nucleated cell count was 2480/mm^3^, with 91% lymphocytes and 7% neutrophils. Acid-fast bacilli smear was negative, while routine bacterial culture showed no growth and cytological examination revealed no malignant cells. The main pleural fluid findings are summarized in Table 2.

The pleural fluid and cervical abscess specimens were tested using Xpert MTB/RIF, which detected *Mycobacterium tuberculosis* complex DNA, with no rifampicin resistance detected in either specimen. Mycobacterial cultures subsequently became positive after 45 days of incubation and confirmed the presence of *Mycobacterium tuberculosis* complex. Extended phenotypic drug susceptibility testing was subsequently performed and demonstrated susceptibility to all first-line antituberculosis drugs tested, with no drug resistance identified.

The Xpert MTB/RIF assay detected *Mycobacterium tuberculosis* complex DNA in both pleural fluid and cervical abscess specimens, with rifampicin resistance not detected. Routine bacterial cultures of both specimens remained sterile. Subsequent mycobacterial cultures confirmed the growth of *Mycobacterium tuberculosis* complex. Given the positive mycobacterial cultures, extended phenotypic drug susceptibility testing was subsequently performed and demonstrated susceptibility to all antituberculosis drugs tested, with no drug resistance identified. Based on the microbiological and susceptibility results, standard first-line antituberculosis therapy with isoniazid, rifampicin, pyrazinamide, and ethambutol was initiated and subsequently continued.

**Figure 4:**
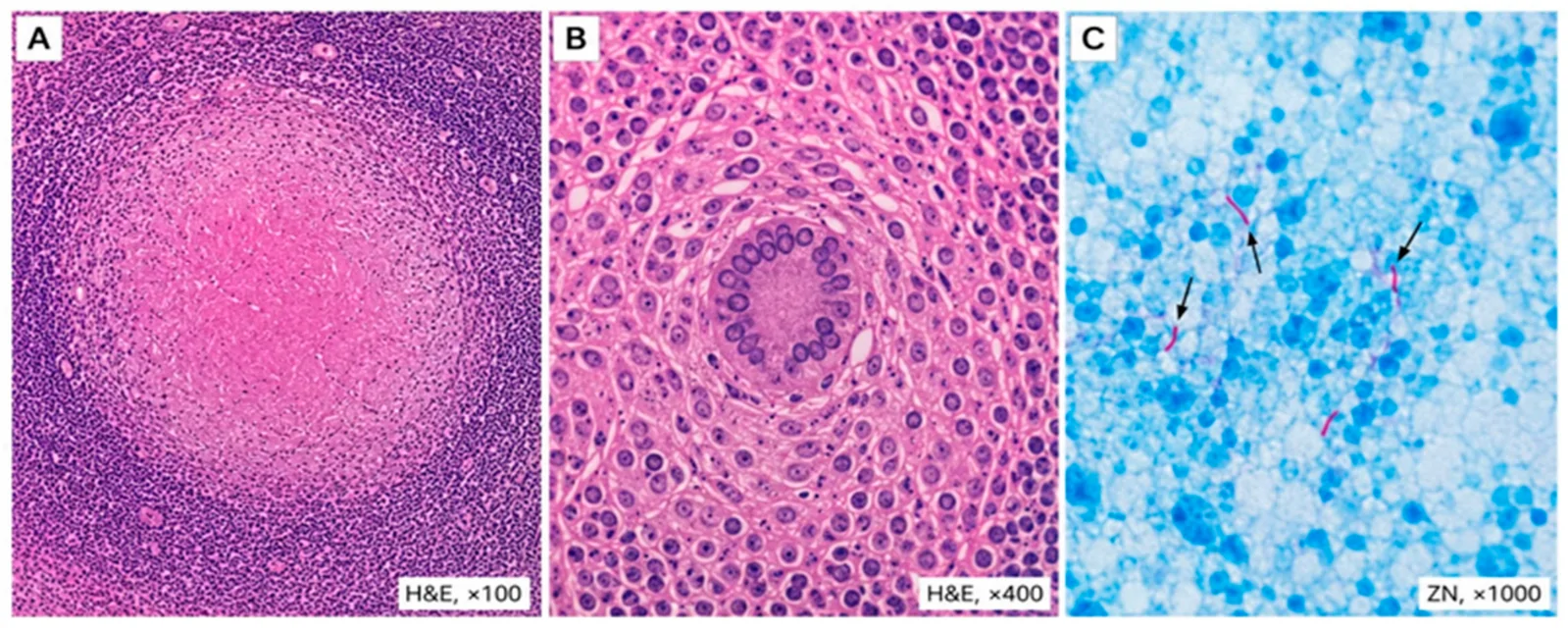
Histopathological findings of tuberculous cervical lymphadenitis. (**A**) H&E staining at ×100 magnification showing granulomatous inflammation with central caseous necrosis; (**B**) H&E staining at ×400 magnification demonstrating a Langhans-type multinucleated giant cell surrounded by epithelioid histiocytes and inflammatory cells; (**C**) Ziehl–Neelsen staining at ×1000 magnification showing occasional acid-fast bacilli (arrows). These findings support the diagnosis of tuberculous cervical lymphadenitis.

Histopathological examination of the cervical tissue demonstrated extensive necrotizing granulomatous inflammation characterized by central caseous necrosis, epithelioid histiocytes, and multinucleated Langhans giant cells, findings highly suggestive of tuberculous lymphadenitis. Ziehl–Neelsen staining additionally demonstrated occasional acid-fast bacilli within the necrotic areas, further supporting the diagnosis of *Mycobacterium tuberculosis* infection (Figure 4).

### 2.4. Treatment and Clinical Outcome

Immediately after molecular confirmation of tuberculosis, standard first-line antituberculous therapy consisting of isoniazid, rifampicin, pyrazinamide, and ethambutol was initiated.

The patient showed rapid clinical improvement following drainage procedures and initiation of antimicrobial therapy. Fever resolved within the first week of treatment, while dyspnea progressively improved as the pleural effusion resolved. The cervical swelling gradually regressed without recurrence or fistula formation (Figure 4).

**Figure 5:**
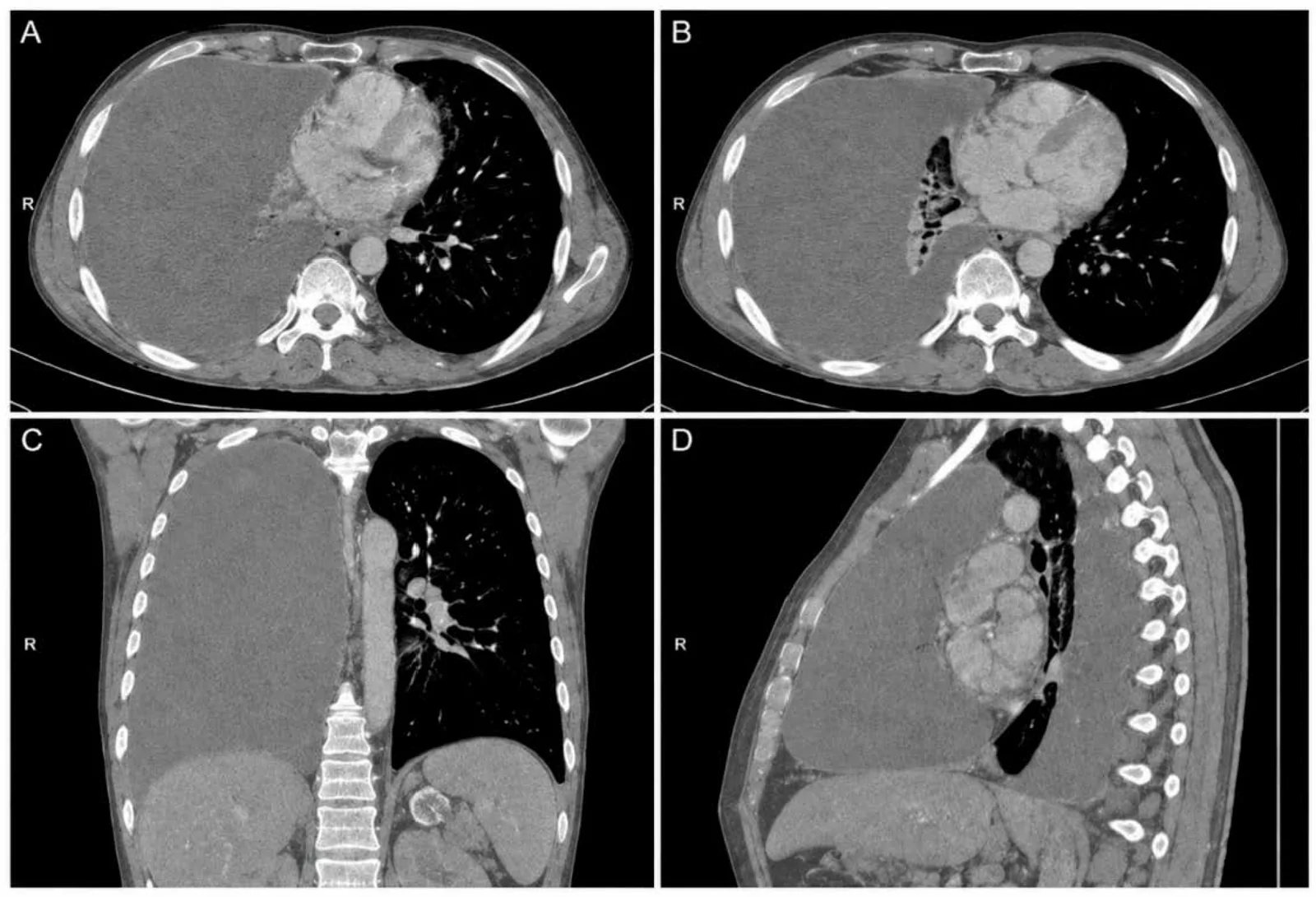
Chest computed tomography (CT) findings of the massive right-sided pleural effusion. (**A**,**B**) Axial CT images demonstrate a large pleural fluid collection causing near-complete opacification of the right hemithorax and substantial compressive atelectasis of the underlying lung, with associated mediastinal shift. (**C**) Coronal reconstruction demonstrates the extensive craniocaudal extent of the pleural effusion, with marked compression of the right lung. (**D**) Sagittal reconstruction further demonstrates the large volume of pleural fluid occupying the right hemithorax, with marked compression of the adjacent right lung. These findings support the radiological characterization of the effusion as massive.

Serial chest radiographs demonstrated progressive re-expansion of the right lung and complete resolution of the pleural effusion during follow-up. At the two-month outpatient evaluation, the patient remained clinically stable, with significant regression of cervical lymphadenopathy, normalization of inflammatory markers, and no evidence of treatment-related adverse effects. Microbiological follow-up confirmed an appropriate response to therapy, and continuation of treatment with isoniazid and rifampicin was planned according to national and international tuberculosis guidelines (Figure 5).

The chronological sequence of diagnostic investigations and therapeutic interventions is summarized in Table 1, highlighting the rapid molecular confirmation of tuberculosis from two independent extrapulmonary specimens and the favorable response following early initiation of specific therapy.

The pleural fluid was classified as an exudate according to Light’s criteria. The pleural fluid protein level was 5.2 g/dL, compared with a serum protein level of 7.6 g/dL, resulting in a pleural fluid-to-serum protein ratio of 0.68 (>0.5). The pleural fluid LDH level was 684 U/L, compared with a serum LDH level of 382 U/L, resulting in a pleural fluid-to-serum LDH ratio of 1.79 (>0.6). Furthermore, the pleural fluid LDH level was higher than two-thirds of the upper limit of normal serum LDH (2/3 × 220 = 146.7 U/L). Thus, all three Light’s criteria for an exudative effusion were fulfilled.

Due to the massive pleural effusion, a chest tube was inserted for therapeutic drainage. Over the following 41 days, a total of seven pleural fluid drainage procedures were performed through the chest tube, with a cumulative volume of 15.7 L evacuated. The volume drained during each procedure was adjusted according to the patient’s clinical tolerance and the amount of pleural fluid present.

In this report, the term “massive pleural effusion” was used to describe a pleural effusion causing near-complete opacification of the affected hemithorax on chest imaging, with substantial compression of the underlying lung, consistent with definitions used in the literature.

Antituberculosis treatment was initiated with a standard four-drug regimen consisting of isoniazid (300 mg/day), rifampicin (600 mg/day), pyrazinamide (1600 mg/day), and ethambutol (1200 mg/day), with doses adjusted according to the patient’s body weight of 72 kg and in accordance with national and international tuberculosis treatment recommendations. The intensive phase was followed by a continuation phase with isoniazid and rifampicin. The total duration of antituberculosis treatment was 8 months.

The patient showed a rapid clinical response after treatment initiation. Fever resolved within the first week, dyspnea progressively improved, and the left cervical swelling regressed. From the third month of treatment, serial clinical examinations documented vesicular breath sounds throughout both pulmonary fields, without persistent clinical evidence of pleural fluid. The patient was monitored periodically throughout treatment through the Tuberculosis Dispensary in Arad, including clinical and laboratory follow-up. No significant treatment-related adverse effects were reported.

## 3. Discussion

The diagnosis of extrapulmonary tuberculosis remains a significant clinical challenge, particularly when the disease presents with atypical manifestations and involves multiple anatomical sites. In the present case, the simultaneous occurrence of tuberculous cervical lymphadenitis (scrofula) and massive pleural tuberculosis represented a rare diagnostic scenario, as both manifestations may develop independently and frequently mimic other infectious, inflammatory, or malignant conditions. The absence of pulmonary parenchymal lesions and the predominance of extrapulmonary involvement further complicated the initial diagnostic approach [14,15,16].

In this case, the diagnosis of extrapulmonary tuberculosis involving multiple non-contiguous anatomical sites was based on microbiological confirmation of *Mycobacterium tuberculosis* complex from two distinct extrapulmonary specimens, namely the cervical abscess and pleural fluid, in association with cervical and mediastinal lymphadenopathy and extensive pleural involvement. Although this pattern is compatible with disseminated extrapulmonary tuberculosis as defined in some clinical and epidemiological studies, hematogenous dissemination was not directly demonstrated in our patient, and no miliary pulmonary pattern was identified on imaging. Therefore, the term “disseminated” is used to describe the involvement of multiple non-contiguous extrapulmonary sites rather than to imply proven hematogenous dissemination [10].

Tuberculous cervical lymphadenitis is the most frequent form of lymph node tuberculosis and represents one of the most common manifestations of extrapulmonary tuberculosis. Clinically, it is characterized by slowly progressive cervical lymph node enlargement, often with minimal systemic symptoms. However, in advanced stages, affected nodes may undergo caseous necrosis, liquefaction, and abscess formation, producing a clinical picture resembling bacterial suppurative lymphadenitis. In our patient, the presence of a painful fluctuant cervical mass initially raised the possibility of a pyogenic cervical abscess; however, the coexistence of constitutional symptoms, weight loss, night sweats, and pleural involvement suggested a more complex infectious process requiring further investigation [17,18,19].

The differential diagnosis of cervical necrotizing lymphadenitis is broad and includes bacterial infections, atypical mycobacterial infections, lymphoma, metastatic malignancies, sarcoidosis, and other granulomatous diseases. Therefore, reliance solely on clinical appearance or routine laboratory parameters may lead to diagnostic delay. Histopathological examination remains an essential component of the diagnostic evaluation, particularly when lymph node tissue is available. In our case, the identification of necrotizing granulomatous inflammation with caseous necrosis, epithelioid histiocytes, and Langhans-type multinucleated giant cells provided strong evidence of tuberculous lymphadenitis. Nevertheless, granulomatous inflammation is not entirely specific for tuberculosis, and microbiological confirmation remains necessary whenever possible [20].

The diagnosis of pleural tuberculosis presents additional difficulties because pleural fluid is typically paucibacillary, resulting in limited sensitivity of conventional diagnostic methods. Acid-fast bacilli smear microscopy from pleural fluid has historically demonstrated low diagnostic yield, often below 10–20%, while mycobacterial culture, although more sensitive, requires several weeks before obtaining results. In the current case, the initial pleural fluid smear was negative, which is consistent with the known limitations of direct microscopy in extrapulmonary disease. However, the lymphocyte-predominant exudative pleural effusion, elevated adenosine deaminase (ADA) level (87 U/L), and concurrent tuberculous lymphadenitis strongly supported the diagnosis of pleural tuberculosis [21,22].

Adenosine deaminase measurement in pleural fluid represents an important diagnostic adjunct in suspected pleural tuberculosis. Increased ADA activity reflects T-cell activation and macrophage response to *Mycobacterium tuberculosis* infection. Although ADA values above 40 U/L are commonly considered suggestive of tuberculosis in appropriate clinical settings, interpretation should always consider local epidemiology and alternative causes of lymphocytic exudative effusions, including lymphoma, malignancy, and autoimmune diseases. In our patient, the markedly elevated ADA level combined with molecular confirmation of *M. tuberculosis* provided strong diagnostic evidence [23,24].

The introduction of nucleic acid amplification tests, particularly the Xpert MTB/RIF assay, has significantly improved the diagnostic pathway of extrapulmonary tuberculosis. Unlike conventional culture methods, GeneXpert provides rapid detection of *M. tuberculosis* complex DNA and simultaneous evaluation of rifampicin resistance within a few hours. Although the sensitivity of Xpert MTB/RIF varies according to specimen type, it has demonstrated superior diagnostic performance when applied to lymph node aspirates compared with pleural fluid. In the present case, the detection of *M. tuberculosis* complex DNA in both cervical abscess material and pleural fluid allowed rapid confirmation of disseminated extrapulmonary tuberculosis and enabled early initiation of specific therapy [25].

The simultaneous molecular confirmation from two independent extrapulmonary sites represents an important diagnostic feature of this case. While GeneXpert positivity in lymph node material is well documented, molecular identification of tuberculosis directly from pleural fluid remains less frequent because of the low bacillary burden. The concordant results obtained from both specimens increased diagnostic confidence and reduced the risk of misclassification as isolated bacterial cervical abscess or nonspecific inflammatory pleuritis [26].

Imaging also played a crucial role in establishing disease extent. Thoracic computed tomography demonstrated massive right-sided pleural effusion with compressive atelectasis and associated mediastinal lymphadenopathy, without evidence of cavitary pulmonary tuberculosis. This pattern highlights that significant thoracic involvement may occur even in the absence of classical pulmonary findings. Cervical ultrasonography further contributed by identifying necrotic lymph nodes with liquefaction, guiding drainage and specimen acquisition for microbiological and histological analysis [27].

An important diagnostic consideration in such cases is the exclusion of immunodeficiency, particularly HIV infection, which significantly increases the risk of extrapulmonary and disseminated tuberculosis. Our patient was HIV-negative and had no identifiable immunosuppressive condition, demonstrating that extensive extrapulmonary disease may also occur in immunocompetent individuals. This finding reinforces the need to consider tuberculosis in the differential diagnosis of complex inflammatory presentations regardless of immune status [24,27].

Although the standard treatment duration for drug-susceptible extrapulmonary tuberculosis is generally 6 months, treatment was continued for a total of 8 months in this patient because of the extensive pleural involvement, the initially massive pleural effusion requiring repeated drainage procedures, and the slow radiological resolution of the pleural disease. Thus, the prolonged treatment duration represented an individualized clinical decision based on the extent of disease and treatment response rather than a routine recommendation for extrapulmonary tuberculosis [7].

Overall, this case emphasizes the need for an integrated diagnostic approach in patients presenting with suppurative cervical lymphadenitis and unexplained pleural effusion, particularly when conventional imaging does not demonstrate pulmonary parenchymal disease. Appropriate extrapulmonary specimens should be evaluated using complementary microbiological, histopathological, and molecular methods [3,15].

## 4. Conclusions

Tuberculous cervical lymphadenitis associated with massive pleural tuberculosis represents an uncommon and diagnostically challenging manifestation of extrapulmonary tuberculosis, particularly in immunocompetent patients without pulmonary parenchymal disease. The combination of suppurative cervical lymphadenitis and a large unexplained pleural effusion should prompt consideration of tuberculosis, even in the absence of classical pulmonary findings [28,29].

In this patient, the diagnosis was established through concordant molecular and microbiological findings from two extrapulmonary sites, supported by characteristic histopathological features. The case highlights the importance of obtaining appropriate specimens for complementary diagnostic testing and of considering tuberculosis early in the evaluation of complex cervical and pleural presentations [30,31].

## Figures and Tables

**Table 1:** Baseline laboratory findings at hospital admission.

Parameter	Result	Reference Range
Hemoglobin (g/dL)	11.8	13.5–17.5
Hematocrit (%)	35.6	40–52
White blood cell count (×10^9^/L)	10.4	4.0–10.0
Neutrophils (%)	72	40–75
Lymphocytes (%)	18	20–45
Platelets (×10^9^/L)	468	150–400
C-reactive protein (CRP) (mg/L)	96.4	<5
Erythrocyte sedimentation rate (ESR) (mm/h)	84	<20
Procalcitonin (ng/mL)	0.18	<0.50
Albumin (g/dL)	3.2	3.5–5.0
Total protein (g/dL)	7.6	6.4–8.3
Lactate dehydrogenase (LDH) (U/L)	382	125–220
Aspartate aminotransferase (AST) (U/L)	28	<40
Alanine aminotransferase (ALT) (U/L)	31	<41
Creatinine (mg/dL)	0.89	0.70–1.30
HIV serology	Negative	Negative

**Table 2:** Pleural fluid analysis.

Parameter	Result
Appearance	Clear yellow (straw-colored)
Protein (g/dL)	5.2
LDH (U/L)	684
Glucose (mg/dL)	78
pH	7.34
Adenosine deaminase (ADA) (U/L)	87
Total nucleated cells (/mm^3^)	2480
Lymphocytes (%)	91
Neutrophils (%)	7
Mesothelial cells (%)	2
Acid-fast bacilli smear	Negative
Routine bacterial culture	No growth
Cytology	Negative for malignant cells
Xpert MTB/RIF (GeneXpert)	Positive for *Mycobacterium tuberculosis*
Rifampicin resistance	Not detected

## Data Availability

The data presented in this study are available on request from the corresponding authors due to privacy and ethical restrictions concerning patient-related clinical information.

## Abbreviations

The following abbreviations are used in this manuscript:

ADA	Adenosine Deaminase
AFB	Acid-Fast Bacilli
CARE	CAse REport Guidelines
CRP	C-Reactive Protein
CT	Computed Tomography
DNA	Deoxyribonucleic Acid
ECDC	European Centre for Disease Prevention and Control
EEA	European Economic Area
EPTB	Extrapulmonary Tuberculosis
ESR	Erythrocyte Sedimentation Rate
H&E	Hematoxylin and Eosin
HIV	Human Immunodeficiency Virus
LDH	Lactate Dehydrogenase
MGIT	Mycobacterial Growth Indicator Tube
MTB	*Mycobacterium tuberculosis*
PCR	Polymerase Chain Reaction
RIF	Rifampicin
TB	Tuberculosis
WHO	World Health Organization
Xpert MTB/RIF	Xpert *Mycobacterium tuberculosis*/Rifampicin Assay
